# Prevalence and Trends of *Staphylococcus aureus* Bacteraemia in Hospitalized Patients in South Africa, 2010 to 2012: Laboratory-Based Surveillance Mapping of Antimicrobial Resistance and Molecular Epidemiology

**DOI:** 10.1371/journal.pone.0145429

**Published:** 2015-12-31

**Authors:** Olga Perovic, Samantha Iyaloo, Ranmini Kularatne, Warren Lowman, Noma Bosman, Jeannette Wadula, Sharona Seetharam, Adriano Duse, Nontombi Mbelle, Colleen Bamford, Halima Dawood, Yesholata Mahabeer, Prathna Bhola, Shareef Abrahams, Ashika Singh-Moodley

**Affiliations:** 1 National Institute for Communicable Diseases at National Health Laboratory Service, Johannesburg, South Africa; 2 Department of Clinical Microbiology and Infectious Diseases, School of Pathology, Faculty of Health Science, University of Witwatersrand, Johannesburg, South Africa; 3 National Health Laboratory Service, Helen Joseph Hospital, Johannesburg, South Africa; 4 National Health Laboratory Service Charlotte Maxeke Johannesburg Academic Hospital Laboratory Complex, Johannesburg, South Africa; 5 National Health Laboratory Service, Chris Hani Baragwanath Academic Hospital, Johannesburg, South Africa; 6 Department of Medical Microbiology at University of Pretoria, Pretoria, South Africa; 7 National Health Laboratory Service, Groote Schuur Hospital, Cape Town, South Africa; 8 University of Cape Town, Cape Town, South Africa; 9 Department of Medicine, Greys Hospital and Caprisa, Pietermaritzburg, South Africa, University of KwaZulu Natal, Durban, South Africa; 10 National Health Laboratory Service, Inkosi Albert Luthuli Central Hospital, Durban, South Africa; 11 School of Laboratory Medicine and Medical Sciences, University of KwaZulu-Natal, Durban, South Africa; 12 National Health Laboratory Service, Stellenbosch, Stellenbosch University, Stellenbosch, South Africa; Amphia Ziekenhuis, NETHERLANDS

## Abstract

**Introduction:**

We aimed to obtain an in-depth understanding on recent antimicrobial resistance trends and molecular epidemiology trends of *S*. *aureus* bacteraemia (SAB).

**Methods:**

Thirteen academic centres in South Africa were included from June 2010 until July 2012. *S*. *aureus* susceptibility testing was performed on the MicroScan Walkaway. Real-time PCR using the LightCycler 480 II was done for *mec*A and *nuc*. SCC*mec* and *spa*-typing were finalized with conventional PCR. We selected one isolate per common *spa* type per province for multilocus sequence typing (MLST).

**Results:**

*S*. *aureus* from 2709 patients were included, and 1231 (46%) were resistant to methicillin, with a significant decline over the three-year period (p-value = 0.003). Geographical distribution of MRSA was significantly higher in Gauteng compared to the other provinces (P<0.001). Children <5 years were significantly associated with MRSA with higher rates compared to all other age groups (P = 0.01). The most prevalent SCC*mec* type was SCC*mec* type III (531 [41%]) followed by type IV (402 [31%]). *Spa*-typing discovered 47 different *spa*-types. The five (87%) most common *spa-*types were t037, t1257, t045, t064 and t012. Based on MLST, the commonest was ST612 clonal complex (CC8) (n = 7) followed by ST5 (CC5) (n = 4), ST36 (CC30) (n = 4) and ST239 (CC8) (n = 3).

**Conclusions:**

MRSA rate is high in South Africa. Majority of the isolates were classified as SCC*mec* type III (41%) and type IV (31%), which are typically associated with hospital and community- acquired infections, respectively. Overall, this study reveals the presence of a variety of hospital-acquired MRSA clones in South Africa dominance of few clones, *spa* 037 and 1257. Monitoring trends in resistance and molecular typing is recommended to detect changing epidemiological trends in AMR patterns of SAB.

## Introduction

The prevalence of antimicrobial resistant bacteria from healthcare-associated infections (HAIs) is increasing [1]. Laboratory-based surveillance for antimicrobial resistance (AMR) generates reliable data on the occurrence of AMR in different geographical regions and provides a platform for future interventions. Amongst pathogens causing hospital infections, Gram-positive cocci have become predominant over the past few decades, globally [2].

The increasing numbers of antimicrobial resistant Gram-positive isolates from hospital settings is demonstrated in the national surveillance reports from high, middle and low income countries [3,4]. However, South Africa has a complex healthcare system; therefore, a national surveillance report of this nature was not available.

*Staphylococcus aureus* is one of the most common Gram-positive pathogens isolated from humans [5]. Importantly, in the last two decades there have been increased reports of methicillin-resistant *S*. *aureus* (MRSA) isolates from the community which were previously only cultured from hospitalized patients.

The prevalence of MRSA in both hospital and community settings varies depending on geographical diversity setting [6]. The changing epidemiology of MRSA is due to the co-existence of healthcare-associated MRSA (HA-MRSA) and community-associated MRSA (CA-MRSA) infections. However, the epidemiology is more complex since some community onset MRSA infections are caused by hospital-associated strains as a result of outpatient management of MRSA infections with so called feral, HA-MRSA strains [5]. This epidemiology of *S*. *aureus* has not been ascertained in the South African setting. *S*. *aureus* bacteraemias (SAB) are difficult to treat and is associated with 29–63% mortality [7,8]. The emergence of new CA-MRSA strains in the community has huge implications on patient treatment [5]. These strains have been distinguished by molecular characterization of the staphylococcal cassette chromosome *mec* (SCC*mec*) with HA-MRSA carrying a large SCC*mec* type I-III and the CA-MRSA strains carrying the smaller SCC*mec* elements IV-V [5]. A number of virulence factors have been identified in these strains such as the Panton-Valentine leukocidin (PVL). Other molecular typing methods such as s*pa*-typing and multilocusmulti-locus sequence typing (MLST) are useful in investigating evolutionary relationships amongst isolates and enable study of routes of transmission to assess the source of infection. Geographically, these isolates are diverse and typing methods are used to confirm common strains in specific regions. It is important to detect and understand the molecular characteristics of MRSA isolates as this may impact on patient’s treatment and antibiotic choice.

### Aims

We aimed to determine the antimicrobial resistance trends and molecular epidemiology of *S*. *aureus* bacteraemia (SAB), in hospitalised South African patients through national laboratory-based sentinel site surveillance over a three year period.

## Methods

AMR Surveillance is a subset of the GERMS-SA (Group for Enteric, Respiratory and Meningeal Surveillance in South Africa) programme which collects isolates for phenotypic and molecular analysis from sentinel sites.

### Patient selection

*S*. *aureus* isolated from blood cultures between June 2010 and July 2012 were received from thirteen academic centres serving the public healthcare sector in SA. The sites represented 4 regions: Gauteng [GP], KwaZulu-Natal [KZN], Free State [FS], and Western Cape [WC] provinces.

### Case definitions

We defined a case of *S*. *aureus* bacteraemia (SAB) as *S*. *aureus* isolated from a blood culture. A new case of SAB in the same patient was reported if the organism was isolated 21 or more days from the date of the first positive blood culture. The term methicillin-resistant *Staphylococcus aureus* (MRSA) defined all oxacillin-resistant isolates.

### Phenotypic methods

*S*. *aureus* isolates were submitted on Dorset transport media (Department of Media Production at NHLS). Organism identification was confirmed using the Vitek 2 GP card (Biomerieux, France). Susceptibility testing was performed on the MicroScan Walkaway system (Siemens Healthcare Diagnostics, USA) using Positive MIC Panel Type 33, with breakpoint ranges from resistant to susceptible. Categorical results and the susceptibility profiles of each antimicrobial agent tested were based on the Clinical Laboratory Standards Institute (CLSI) interpretative criteria [9]; Breakpoint tables for interpretation of MICs and zone diameters by EUCAST [10] and/or the MicroScan recommendations. The MIC_50_ and MIC_90_ (minimum inhibitory concentrations needed to inhibit the growth of 50% and 90% of organisms, respectively) were determined.

### Molecular methods

#### DNA Extraction

Half a loop of bacterial culture from purity plates was re-suspended in 400μl TE buffer. This was vortexed briefly and heated at 95°C for 25 min to allow bacterial cell lysis to release the DNA. Centrifugation followed at 12000rpm for 3 min to pellet the cellular debris. The supernatant was then aliquoted and stored at -70°C for further use.

#### Polymerase Chain Reaction (PCR) screening for *mecA* and *mecC* genes in MRSA isolates

The LightCycler 480 II (Roche Applied Science) instrument was used for the real-time PCR of *mecA* and *nuc* which were amplified in a multiplex assay using the LightCycler 480 Probes Master kit (Roche Diagnostics, IN, USA) with previously published primers and probes [11]. The G-Storm (Somerton Biotechnology Centre, UK) thermal cycler was used for the conventional PCR of *mecC* using the Qiagen Multiplex PCR kit (Qiagen, Germany) with previously published primers [12].

#### SCC*mec* typing

All *mecA*-positive MRSA isolates were typed by multiplex PCR using the Qiagen Multiplex PCR kit (Qiagen, Germany) and previously published primers [13].

#### *Spa*-typing

Spa-typing was performed on 569 MRSA isolates. The spa gene was amplified using previously published primers [14] and the Amplitaq Gold DNA Polymerase kit (Applied Biosystems, CA, USA). Purified PCR products (Qiagen Purification kit; Qiagen, Germany) were sequenced (Inqaba Biotech, South Africa). Sequences were assembled using CLC Bio main workbench (Qiagen, Germany) and analysed using the Ridom StaphType^™^ software (Ridom GmbH, Würzburg, Germany).

#### Multilocus sequence typing (MLST)

One isolate per common *spa* types per province were selected for MLST. Primers [15] amplifying seven reference genes were used. Amplification was done using the Amplitaq Gold DNA Polymerase kit (Applied Biosystems, CA, USA). Purified PCR products were sequenced (Inqaba Biotech, South Africa). Sequences were assembled using CLC Bio main workbench (Qiagen, Germany) and analysed using the online database (http://saureus.mlst.net/).

### Ethics

Approval for this retrospective study was obtained from the Human Research Ethics Committee (Medical) (HREC), University of Witwatersrand, Johannesburg (protocol number M10464). All patients information were anonymized preceding the analysis.

### Statistical analysis

We used the Pearson’s chi-squared test or Fisher’s exact test as applicable to calculate *P*-values for analysis of trends in antibiotic susceptibility. A *P*-value <0.05 was deemed statistically significant.

## Results

**Fig 1:**
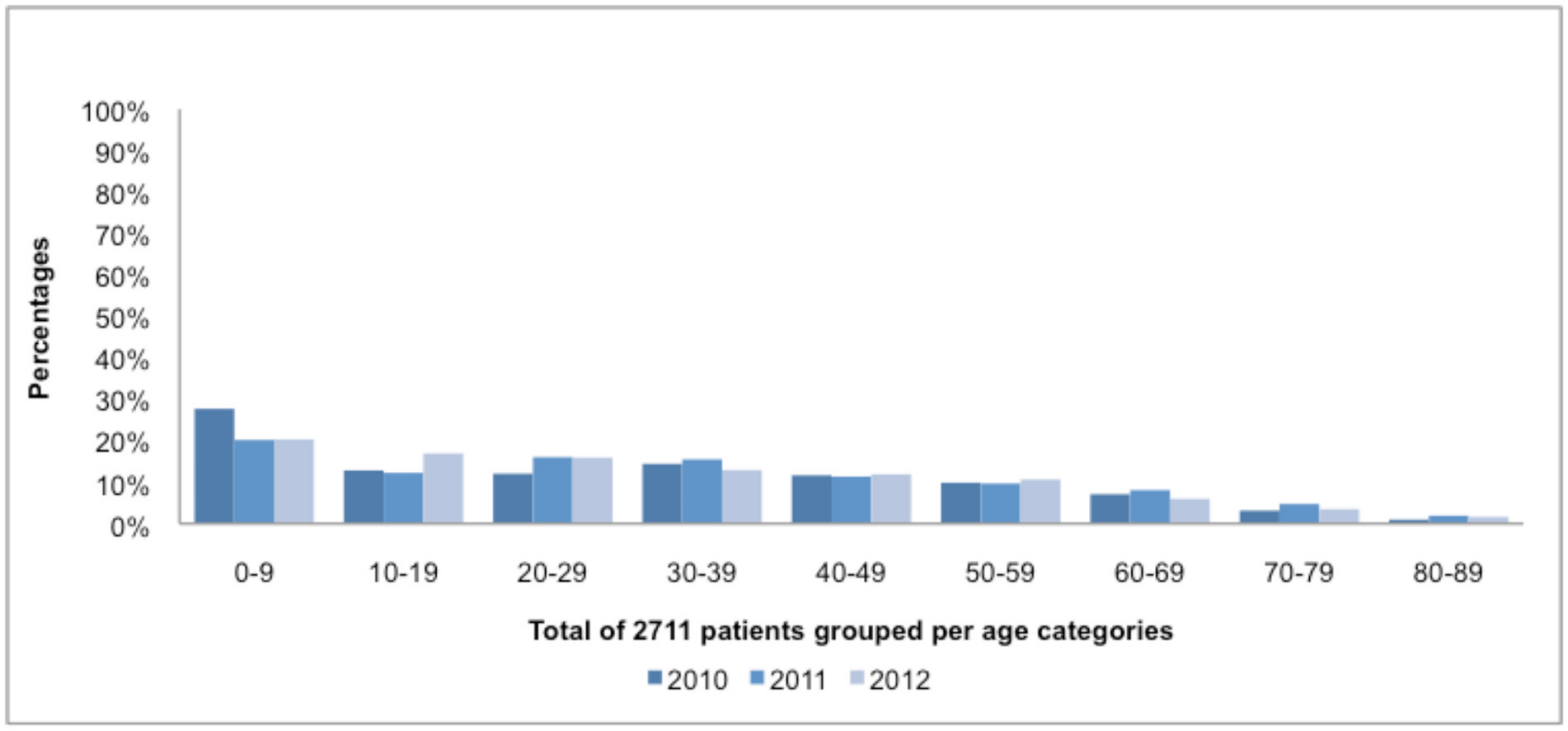
Age distribution of 2711 patients with SA bacteraemia from June 2010—July 2012.

A total of 2709 *S*. *aureus* isolates from patients with bacteraemia were included during the study period. The majority of patients were adult males (51%). A higher prevalence of SAB was found in young patients (0–9 yearage group) (33%) in Fig 1. The highest numbers of isolates were from patients in Gauteng, 1612 (59.5%).

**Fig 2:**
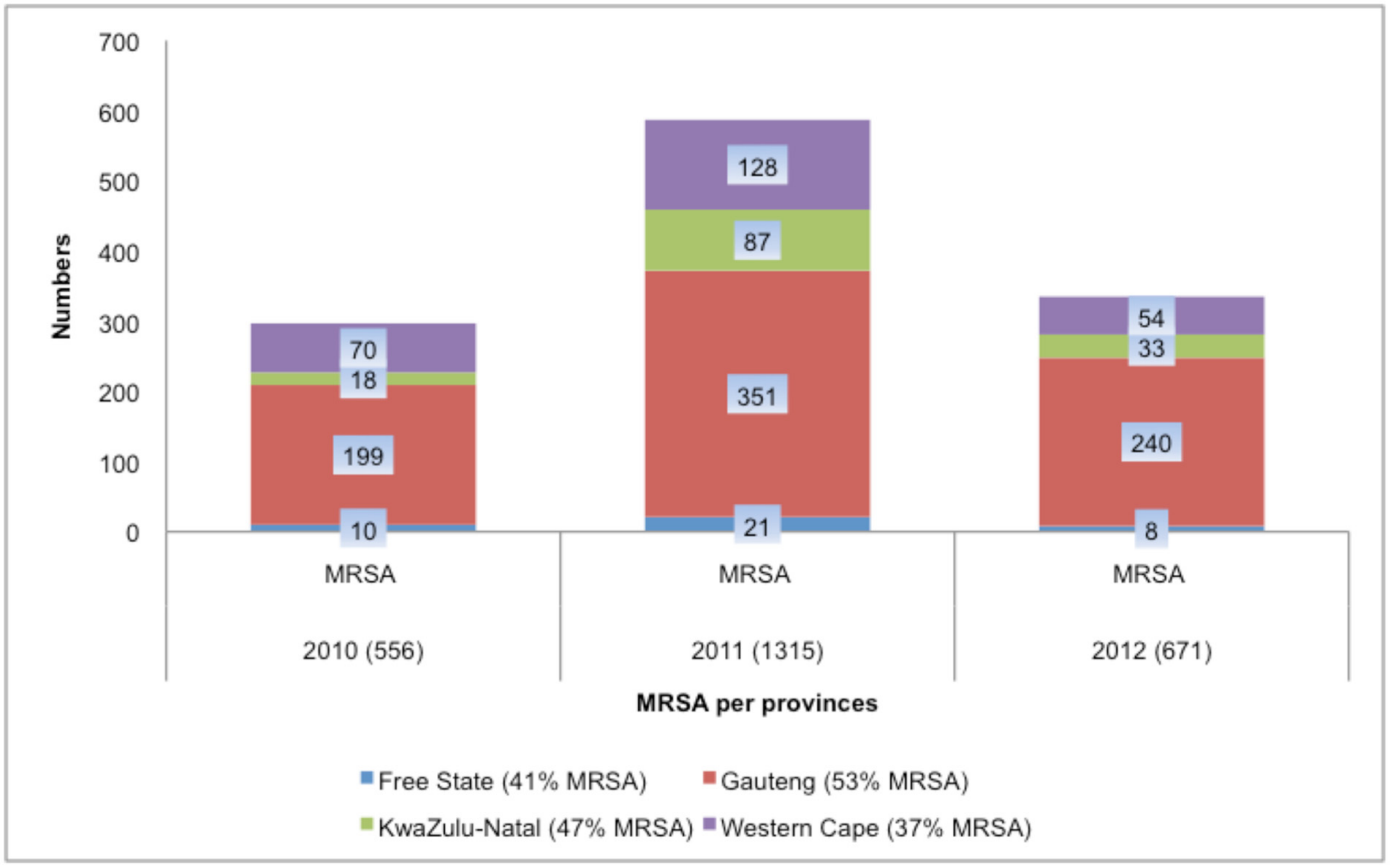
Number of MRSA isolates from four provinces.

**Fig 3:**
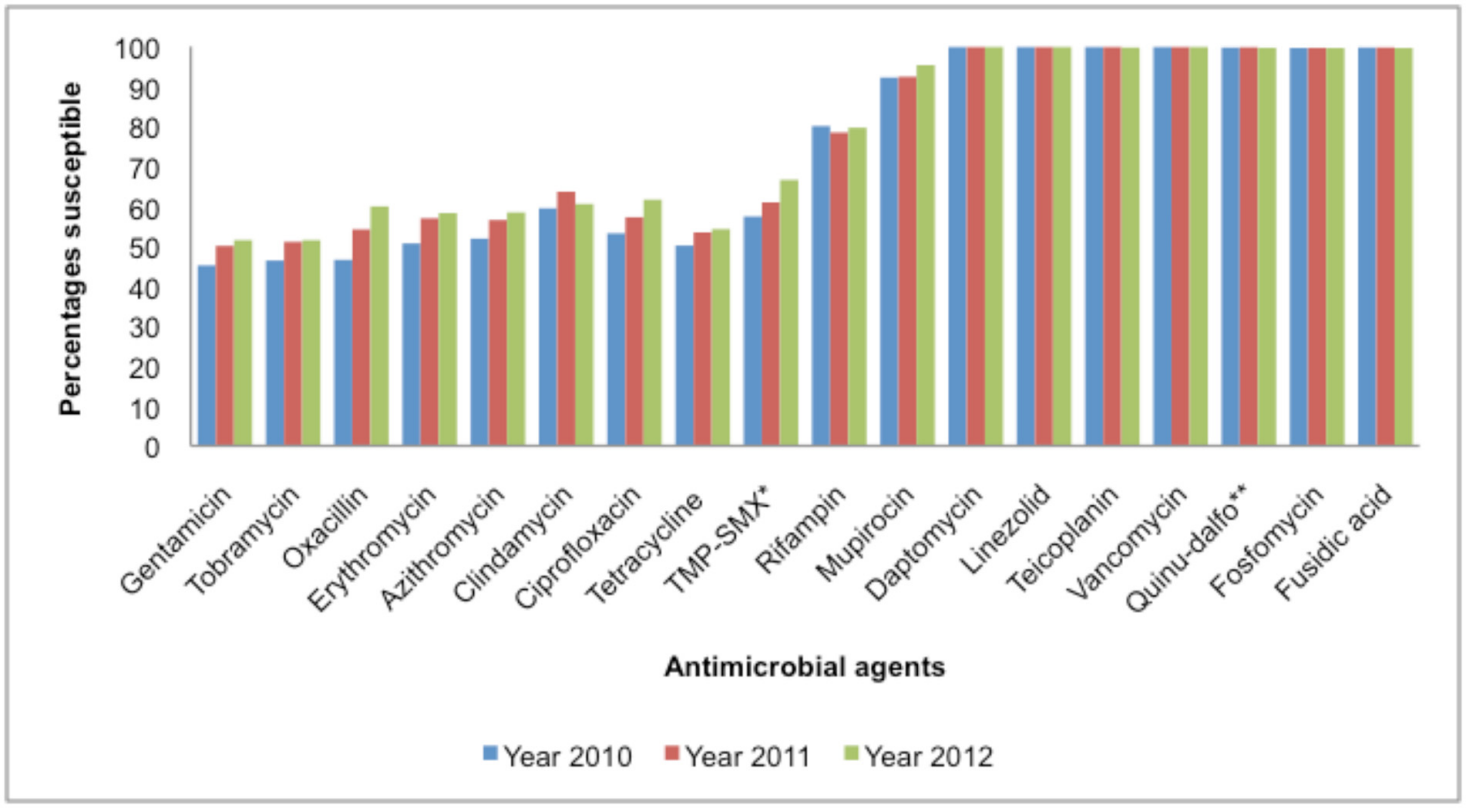
Antimicrobial susceptibility of 2709 SA isolates.

Methicillin resistance was detected in 1231 (46%) isolates with the MIC method (Fig 2) and confirmed with *mec*A in 1160 (43%) isolates (3% discrepant results). During the study period, MRSA rates declined significantly from 53% in 2010 to 40% in 2012 (P = <0.001) (Fig 3). Resistance to macrolides, aminoglycosides, tetracycline, rifampin and mupirocin remained comparable while ciprofloxacin and trimethoprim-sufamethoxazole resistance significantly declined over the surveillance period (P = 0.003 and 0.001 respectively) (Fig 3). MIC_50_ and MIC_90_ remained stable for all antimicrobials tested with no changes over the study period (Table 1).

**Table 1:** Antibiotics MIC_50_, MIC_90_ and breakpoints for 2 709 *S*. *aureus* isolates.

Antibiotics	2010 (total number of isolates 558)		2011 (total number of isolates 1315)		2012 (total number of isolates 836)		MIC interpretative breakpoints (μg/ml) based on CLSI	
	MIC_50_	MIC_90_	MIC_50_	MIC_90_	MIC_50_	MIC_90_	S	R
Gentamicin	≤4	>8	≤4	>8	≤4	>8	≤4	≥16
Tobramycin	8	>8	≤4	>8	≤4	>8	≤4	≥16
Oxacillin	≤2	>2	≤2	>2	≤2	>2	≤2	≥4
Erythromycin	≤0.5	>4	≤0.5	>4	≤0.5	>4	≤0.5	≥8
Azithromycin	≤2	4	≤2	4	≤2	4	≤2	≥8
Clindamycin	≤0.25	0.5	≤0.25	0.5	≤0.25	0.5	≤0.5	≥4
Daptomycin	≤1	≤1	≤1	≤1	≤1	≤1	≤1	-
Ciprofloxacin	≤1	>2	≤1	>2	≤1	>2	≤1	≥4
Tetracycline	≤4	>8	≤4	>8	≤4	>8	≤4	≥16
Rifampin	≤1	>2	≤1	>2	≤1	>2	≤1	≥4
Linezolid	2	2	2	2	2	2	≤4	≥8
Trimethoprim/sulfamethoxazole	≤2/38	>4/76	≤2/38	>4/76	≤2/38	>4/76	≤2/38	≥4/76
Mupirocin	≤4	256	≤4	256	≤4	256	≤4	≥256
Teicoplanin	≤1	≤1	≤1	≤1	≤1	≤1	≤8	≥32
Vancomycin	1	1	1	1	1	1	≤2	≥16
Quinupristin-dalfopristin	≤1	≤1	≤1	≤1	≤1	≤1	≤1	≥4
Fosfomycin*	≤32	≤32	≤32	≤32	≤32	≤32	≤32	≥32
Fusidic acid**	≤2	≤2	≤2	≤2	≤2	≤2	≤2	≥32

* Based on EUCAST

**Based on Comite de Antibiogramme de la Societe Francaise de Microbiologie (CA-SFM, 2008).

**Fig 4:**
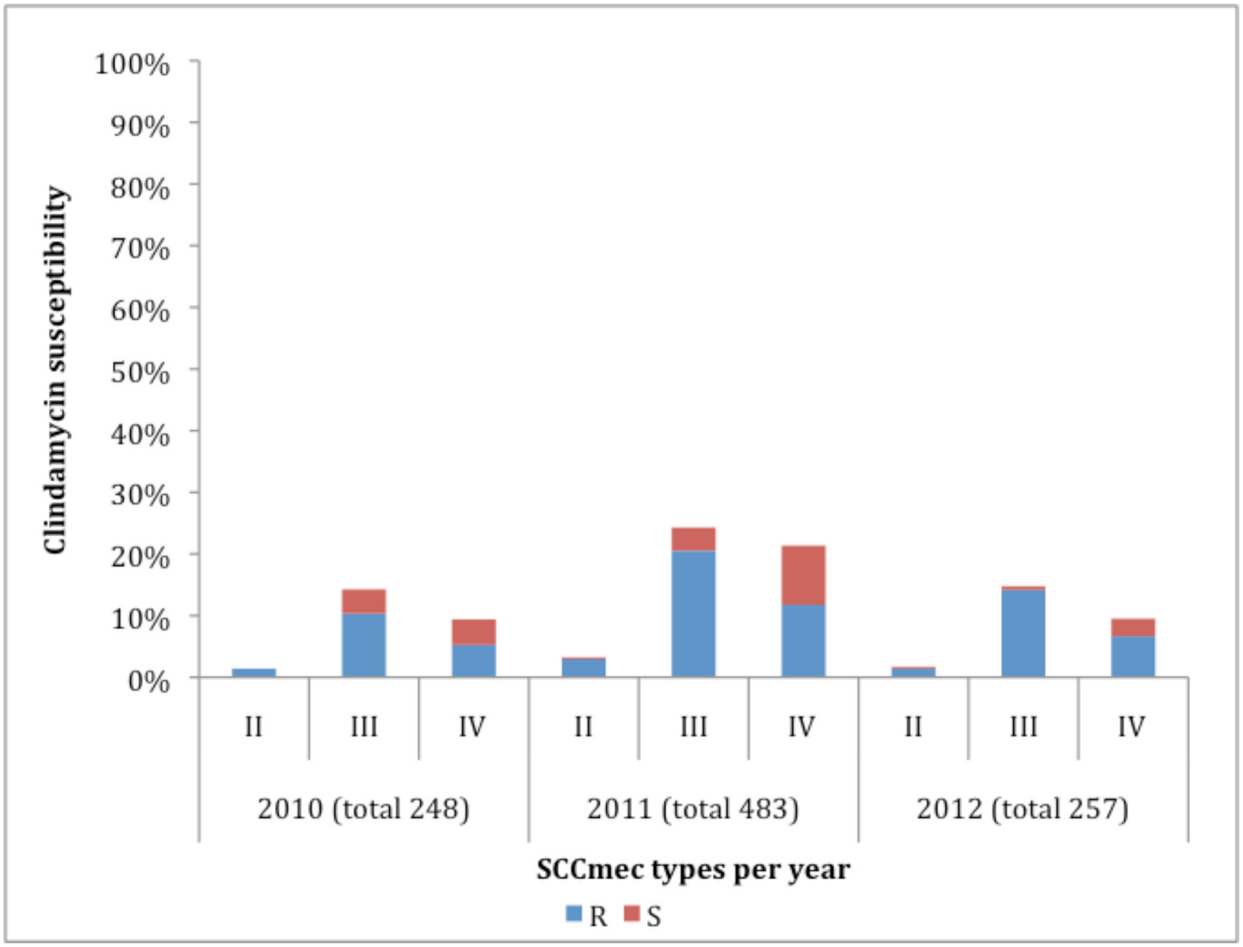
Association between the most common SCC*mec* types and clindamycin susceptibility (n = 988).

MRSA was resistant to more of the other classes of antimicrobial agents compared to MSSA (Table 2). Isolates remained fully susceptible to glycopeptides, daptomycin, linezolid, quinupristin-dalfopristin throughout the study period (Fig 3). From 988 SCC*mec* types (II-IV), 75% were clindamycin resistant and 59% of these isolates were healthcare associated SCC*mec* types II and III (Fig 4).

**Table 2:** Comparison of MRSA and MSSA in susceptibility to antimicrobial agents for 2709 *S*. *aureus* isolates.

Antimicrobial agents	MSSA		MRSA		P-VALUE
	Susceptible (%)	Non-susceptible (%)	Susceptible (%)	Non-susceptible (%)	
Amikacin	19.7	80.34	6.6	93.4	*0*.*004*
/Augmentin	98.5	1.5	0.1	99.9	*<0*.*001*
Ampicillin	10.5	89.5	0	100	<0.001
Azithromycin	89.5	10.5	16.3	83.7	<0.001
Cefepime	99.3	0.7	0.1	99.9	<0.001
Cefoxitin	99.8	0.2	5.1	94.9	<0.001
Cefuroxime	98.9	1.1	0.1	99.9	<0.001
Ciprofloxacin	94	6	15.2	84.8	<0.001
Clindamycin	92.9	7.1	24.8	75.2	<0.001
Erythromycin	89.4	10.6	16.2	83.8	<0.001
Fosfomycin	99.6	0.4	99.6	0.4	0.818
Fusidic Acid	99.4	0.6	98.1	1.9	0.002
Gentamicin	83.8	16.2	8.3	91.7	<0.001
Linezolid	100	0	100	0	N/A
Mupirocin	97.8	2.2	82.6	17.4	<0.001
Rifampin	94.8	5.2	57.4	42.6	<0.001
Teicoplanin	100	0	100	0	N/A
Vancomycin	100	0	100	0	N/A
Tetracycline	83.8	16.2	16.6	83.4	<0.001
Tobramycin	87.2	12.8	6.5	93.5	<0.001
Moxifloxacin	94.8	5.2	22.1	77.9	<0.001

### PCR Screening for *mec*A and *mec*C in MRSA isolates

All genotypically confirmed MRSA isolates harboured both the *mecA* and the species specific *nuc* genes (except 2 *nuc-*negative isolates). No isolate harboured the *mecC* gene.

#### SCCmec typing

**Fig 5:**
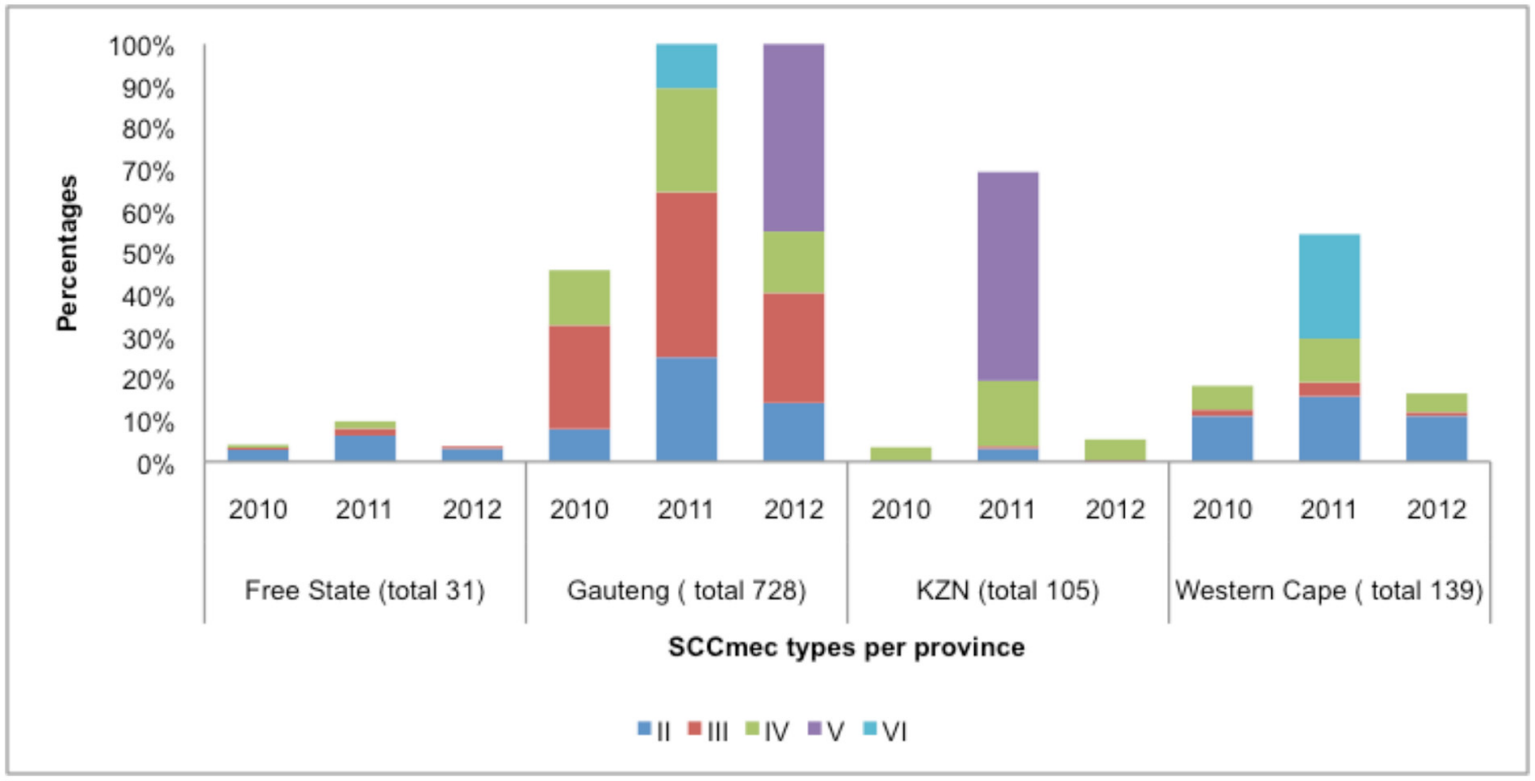
Distribution of 1003 SCC*mec* types over the period of three years per province.

**Fig 6:**
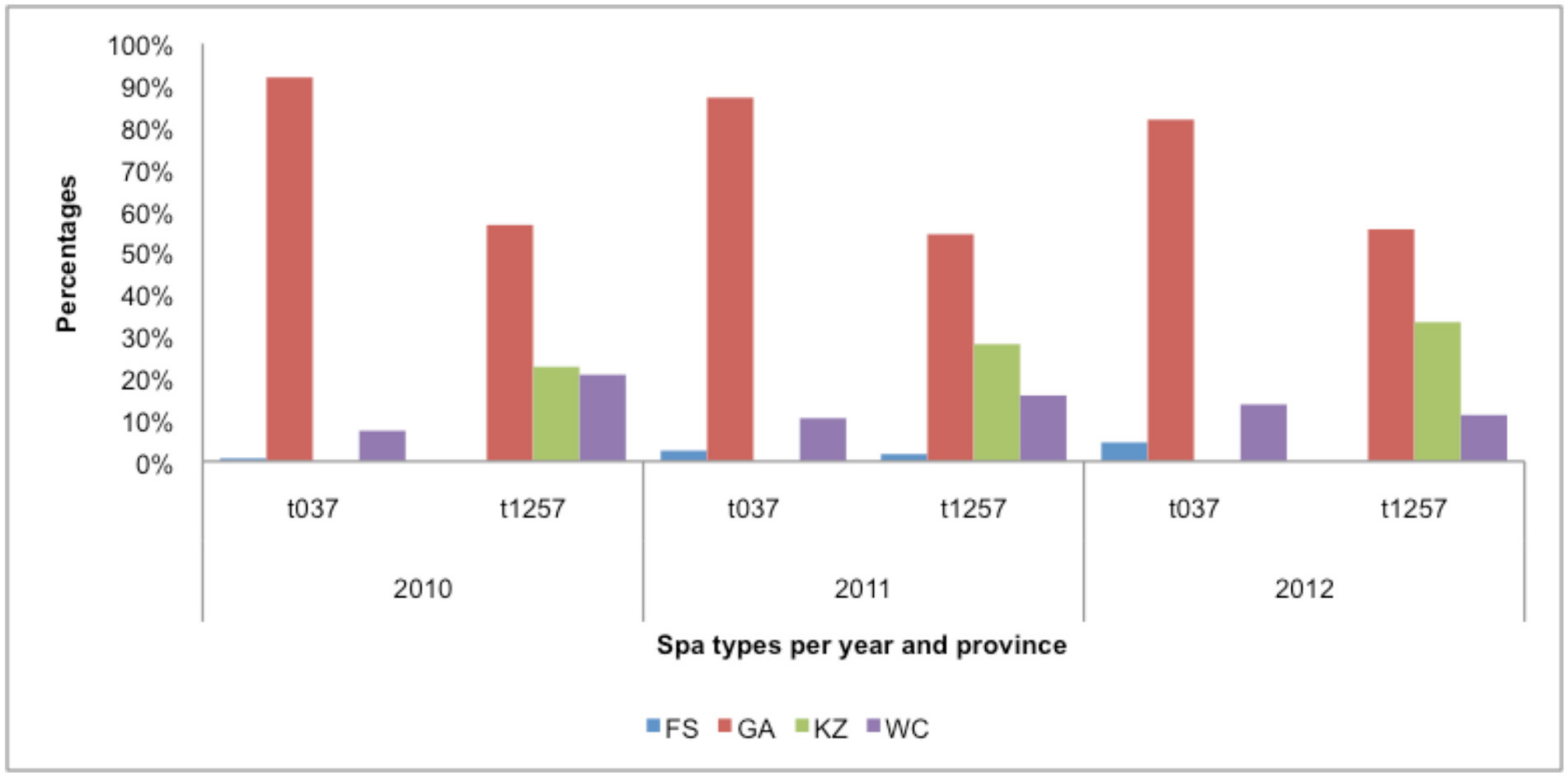
Distribution of the most common 393 *spa*-types per province (total number = 569).

MRSA isolates (1236) were typed to identify the prevalent *mec* element types. Distribution of SCC*mec* types across provinces differed as the numbers of isolates received from each province varied. SCC*mec* types III and IV were predominantly isolated in Gauteng and the emergence of type V was noted in 2012. Type V was identified in KwaZulu Natal in 2011. SCC*mec* type VI was predominantly isolated in Western Cape in 2011. Type IV was found in all provinces. Overall the most prevalent SCC*mec* type for all three years was SCC*mec* type III (531 [41%]) followed by types IV (402 [31%]), II (64 [5%]), VI (4 [0.3%]) and V (2 [0.2%]) (Fig 5). SCC*mec* type I was not observed but unknown typing patterns were identified (185 [12%]). Three isolates produced no amplicons. The majority of isolates representing SCC*mec* types III and IV were from Gauteng [482 (37%) and 212 (16%), respectively] followed by the Western Cape [31 (2%) and 83 (6%), respectively] and KwaZulu-Natal [5 (0.4%] and 97 (8%) respectively] (Fig 6). Of the 185 unknown typing patterns observed, the majority (113, 61%) was from the Western Cape.

#### Spa-typing

Spa-typing of 569 of the isolates revealed 47 different spa-types, nine of which were novel and have not as yet been assigned.

**Fig 7:**
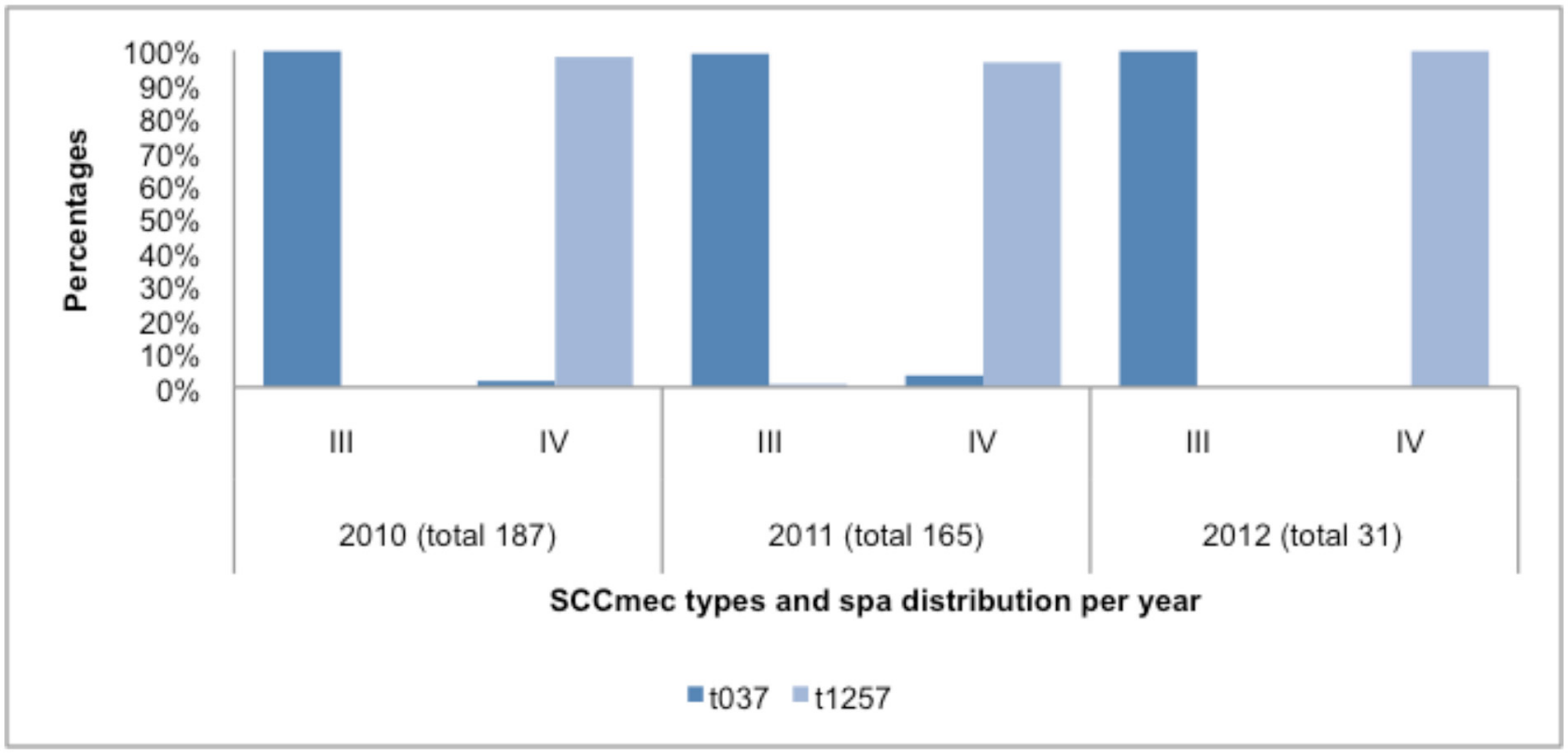
Relationship between the most common SCC*mec* and *spa* types.

The five most common *spa*-types were t037 (n = 274), t1257 (n = 120), t045 (n = 42), t064 (n = 34) and t012 (n = 22) which accounted for 87% of the isolates tested. *Spa*-type was t037 -and t1257 related to hospital- and community- associated infections respectively (Figs 6 and 7). Both were distributed in all four provinces except KZN, where *spa*-type t037 was not found (Fig 6).

The *spa*-types clustered into 4 *spa* clonal complexes (*spa*-CC) using the Based Upon Repeat Pattern (BURP) algorithm at a cost setting of ≤4 and excluding *spa*-types with 5 or fewer repeats (Ridom StaphType^™^ software, Ridom GmbH, Würzburg, Germany). *Spa*-CC-037/012 (54% of all *spa*-types) was the largest clonal complex followed by *spa*-CC-064 (32% of all *spa*-types) and *spa*-CC-045 (7.7% of all *spa*-types). *Spa*-CC-064 contained isolates displaying predominantly the SCC*mec* type IV element. *Spa*-CC-037 contained isolates that displayed SCC*mec* types II, III, IV and unknown typing patterns with a predominance of type III (Fig 3). These clonal complexes were widespread in South Africa. *Spa*-CC-064 and *spa*-CC-037 were identified in Gauteng, Western Cape, KwaZulu-Natal and the Free State provinces.

#### Multilocus sequence typing (MLST)

One isolate belonging to each of the most common *spa*-types per province was selected for MLST. The commonest ST was ST612 of clonal complex (CC8) (n = 7) followed by ST5 (CC5) (n = 4), ST36 (CC30) (n = 4) and ST239 (CC8) (n = 3). One isolate with *spa*-type t1257 produced a new *pta_* allele; therefore a ST could not be assigned. The isolates corresponding SCC*mec* types, *spa*-types and ST can be seen in Table 3.

**Table 3:** Genotypes of 569 MRSA isolates.

Spa-CC and Spa-Types	MLST	SCCmec Type
**spa-CC-064 (n = 183)**		
t008		Unknown
**t064**	ST612 (CC8)	IV
t451		IV
t951		IV
t1257	ST612 (CC8), new ST	IV, II
t1443		IV
t1476		Unknown
t1555		IV
t1852		Negative
t1774		IV
t1971		IV
t2293		IV
t4268		IV
t4833		IV
**spa-CC-037/012 (n = 311)**		
**t012**	ST36 (CC30)	Unknown, II, III
t018		Unknown, II
t021		II
**t037**	ST239 (CC8)	Unknown, II, III, IV
t238		II
t421		III
t932		III
t840		II
t2029		III
t7962		III
**spa-CC045 (n = 44)**		
**t045**	ST5 (CC5)	Unknown
t1107		Unknown
t13165		Unknown
t2724		IV
**No founder identified (n = 6)**		
t022		IV
t032		IV

## Discussion

This study highlights the changing pattern of *S*. *aureus* resistance to oxacillin (MRSA) and other agents using laboratory based sentinel site surveillance data that impacts on patient management. Gauteng province contributed to the majority of isolates (59.5%) and the highest prevalence of MRSA (53%), most likely as it is the most populated province with the largest academic centres. The high percentage of bacteraemic SA isolates that were resistant to oxacillin (46%) is of serious public health concern.

The rate of MRSA from previous studies varies. A systematic review of 263 articles identified on PubMed for the epidemiology of MRSA in Africa showed percentages of MRSA from 12 in Tunisia to 82 in Egypt. Low prevalence of MRSA was 3.7% for all specimens and 6.5% in blood from two private hospitals in Kenya [4,16]. However in 1997, a Kenyan national referral hospital reported a MRSA prevalence of 40%. This significant decrease in MRSA was possibly explained as a result of changing in diagnostic methods or true decline which could be answered by continuous monitoring of AST. During our study period, we identified a decline in the MRSA rate from 53% in 2010 to 40% in 2012 with the exception of Gauteng and a decline in resistance to aminoglycosides and fluoroquinolones which may be linked to the same resistance element. MRSA rate was the lowest in the WC province (37%). This might be explained by geographic variation in the prevalence of organisms in the hospitals across the country. In addition the implementation of the infection prevention and control bundles approach, comprising contact precautions, hand hygiene and a change in institutional practices antibiotic prescription culture advocated both nationally and internationally may have contributed to the possible decline in MRSA rate.

In our study, resistance to clindamycin (40%) was higher than that described by Marais in 2009 [17]. Most of clindamycin resistant isolates were MRSA, SCC*mec* III and IV.

Resistance to mupirocin using EUCAST breakpoints (>256mg/L) ranged from 8% to 5%, which was similar to the 2009 report [15].

Rare cases of reduced susceptibility to glycopeptides were reported in MRSA isolates [5]; in this study all isolates were susceptible.

All strains carried the *mecA* gene, while the *mecC* was absent. Also, *mecC* is associated with specific sequence types not prevalent in South Africa [18]. The majority of isolates were classified as SCC*mec* type III (41%) typically associated with hospital-acquired infections and 31% of isolates harboured the SCC*mec* type IV element commonly associated with community-acquired infections [19–22]. Our findings have indirect impact on patient care and finally on public health overall. Distribution of SCC*mec*, *spa* and MLST types are important for epidemiological understanding of transmission of SA isolates by linking resistance patterns with responding genes. Many data have been published on geographical distribution of MRSA clones mainly from developed countries; our finding will provide South African dynamics. However, since we do not have sufficient epidemiological data we cannot make these conclusions. Interestingly, the prevalence of SCC*mec* type III was higher than SCC*mec* type IV in Gauteng and in the Free State. However, in KwaZulu-Natal and in the Western Cape, SCC*mec* type IV showed predominance over SCC*mec* type III. A prevalence of type IV has been seen previously reported in KwaZuluNatal and the Western Cape [23,24]. Three isolates produced no SCC*mec* type. These isolates may express a type not detected with this method or the SCC*mec* element may have been excised altogether. Primers used in the reaction amplified 10 loci specific for types I to VI. Due to variability and the SCC*mec* element being less conserved in certain regions, detection of regions other than those amplified by our primers may have been omitted, hence the negative result. Furthermore, excision of SCC*mec* does occasionally occur. In the absence of selection pressure SCC*mec*-excised derivatives could arise *in vivo* where fitness and competition play a role. Moreover, the use of certain antibiotics such as vancomycin may lead to spontaneous excision of the element [25].

Further molecular characterisation revealed that *spa*-CC-064 contained isolates displaying predominantly the SCC*mec* type IV element whereas s*pa*-CC-037contained isolates that predominantly displayed SCC*mec* type III. Both these *spa* clonal complexes were identified in Gauteng, Western Cape, KwaZulu-Natal and the Free State. This is in keeping with previous findings in South Africa [24,26,27].

We further grouped these *spa* clonal complexes into three major MLST clonal complexes (CC5, CC8 and CC30). CC8 was the most common and consisted of ST612 and ST239 belonging to s*pa*-CC-064 and s*pa*-CC-037 respectively. *Spa*-CC-037 also consisted of ST36 (CC30) and *spa*-CC-045 consisted of ST5 (CC5). A previous study conducted in South Africa identified the following major clones that are disseminated globally [27]. These were s*pa* type t045-SCC*mec*I-ST5 (CC5), *spa* type t037-SCC*mec*III-ST239 (CC8), *spa* type t012-SCC*mec*II-ST36 (CC30) and *spa* type t064-SCC*mec*IV-ST612 (CC8). In this study we show similar findings with the exception of *spa* type t045-SCC*mec*I-ST5 (CC5) since the SCC*mec* type identified in our study was unknown.

The other clone identified in our study was *spa* type t1257-SCC*mec*IV-ST612 (CC8). *Spa-*types t012 and t037 were grouped together based on *spa* clonal complex (*spa-*CC-037) by BURP analysis. However, these *spa* types clustered independently of each other based on SCC*mec* types (SCC*mec* types II and III respectively) and MLST (ST36 (CC30) and ST239 (CC8) respectively). This was also seen in a previous study where *spa-*types t012 and t037 grouped together based on *spa* clonal complex (*spa-*CC012) but clustered independently of each other based on the other molecular typing methods [27]. The presence of these two distinct MRSA clones is as a result of recombination of a 557kb *spa* fragment from ST30 (CC30) into CC8 in the evolution of ST239 (CC8) [28]. Overall, the most common *spa*-type and ST observed in our study agrees with previous findings in South Africa. ST612 has to date only been described in South Africa and Australia [24,26,27].

Due to the population distribution in our country, the majority (59.5%) of analysed isolates were from Gauteng province followed by the Western Cape (26%), KwaZulu Natal (11%) and the Free State province (3.5%). The limitation of this study was that MLST was performed on few isolates, only. It would have been interesting to compare clones obtained in our study to those observed previously, especially with regards to geographical location. For example, in the study by Moodley *et al*., in 2010, F-*spa*CC-064-SCC*mec*IV-ST612 was not detected in Gauteng province from isolates collected in 2005–2006 [27]. However, although we do not have PFGE results, *spa*CC-064-SCC*mec*IV-ST612 was observed in Gauteng in our study approximately 4 years later, possibly suggesting the transmission or evolution of clones [27]. In addition, we only performed MLST on selected isolates collected from each participating province.

## Conclusions

MRSA rate is high in South Africa. No resistance to glycopeptides, fluoroquinolones, linezolid, daptomycin, synercid and fosfomycin was recorded. Majority of the isolates were classified as SCC*mec* type III (41%) and type IV (31%), which are typically associated with hospital and community- acquired infections, respectively. Overall, this study reveals the presence of a variety of hospital-acquired MRSA clones in South Africa dominance of few clones, *spa* 037 and 1257. Monitoring trends in resistance and molecular typing is recommended to detect changing epidemiological trends in AMR patterns of SAB.
